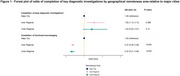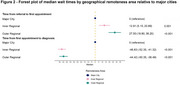# Rural‐urban disparities in the diagnostic pathway of patients with all‐cause mild cognitive impairment or dementia due to Alzheimer's disease in Australia

**DOI:** 10.1002/alz70860_102814

**Published:** 2025-12-23

**Authors:** Xiaoping Lin, Colman Taylor, Elisabeth de Laguiche, Elizabeth Adesanya, Henry Brodaty, Jonathan Pearson‐Stuttard, Mohammad Amin Honardoost, Sara Holloway, Stephanie Alison Ward, Susannah Ahern, Michael Woodward

**Affiliations:** ^1^ Monash University, Melbourne, VIC, Australia; ^2^ Htanalysts, Suite 801, 46 Kippax Street, Surry Hills NSW 2010, Surry Hills, NSW, Australia; ^3^ Novo Nordisk, København S, København S, Denmark; ^4^ Lane Clark & Peacock, London, London, United Kingdom; ^5^ Centre for Healthy Brain Ageing (CHeBA), UNSW Sydney, Sydney, NSW, Australia; ^6^ Department of Continuing Care, Austin Health, The University of Melbourne, Melbourne, VIC, Australia

## Abstract

**Background:**

Existing literature has identified inequalities in incidence and health outcomes between urban and rural populations with Alzheimer's disease (AD), however, potential disparities in the diagnostic process are poorly understood. We aimed to investigate differences in the diagnostic pathway – including the completion of key diagnostic investigations and diagnostic wait times – among individuals with all‐cause mild cognitive impairment (MCI) or dementia due to AD residing in rural and urban Australia.

**Method:**

We conducted a cross‐sectional study using data from the Australia Dementia Network (ADNeT) Registry. Patients diagnosed with all‐cause MCI or AD dementia between registry commencement (March 2020) and December 2023 were included. Participants were categorised into three geographic groups – Major Cities (urban), Inner Regional, Outer Regional (both rural) – based on patient postcode (or clinic postcode if unavailable). Logistic and quantile regression models were used to investigate associations between rural/urban residence and the clinical diagnostic pathway.

**Result:**

We identified 3,648 patients, 1,455(39.88%) with all‐cause MCI and 2,193(60.12%) with dementia due to AD. Participants in inner regional areas were more likely (odds ratio [OR]=1.55; 95% confidence interval [CI]=1.14,2.13; *p* = 0.006) to have had more basic diagnostic investigations completed (including core blood tests, cognitive assessments, functional assessments, structural neuroimaging) compared to those in major cities. However, participants in both inner regional (OR=0.37; 95% CI=0.28,0.48; *p* <0.001) and outer regional (OR=0.32; 95% CI=0.21,0.48; *p* <0.001) areas were less likely to have functional neuroimaging completed. Median wait times for an initial appointment following referral to a memory clinic were up to 28 days longer for rural compared to urban participants (Inner regional: Beta (median)=12.92; 95% CI=5.15,20.69; *p* = 0.001; Outer regional: Beta (median)=27.50; 95% CI=18.80,36.20; *p* <0.001). However, median wait times from initial appointment to diagnosis were up to 47 days shorter in rural compared to urban residents (Inner regional: Beta (median)=‐46.83; 95% CI=‐52.35,‐41.32; *p* <0.001; Outer regional: Beta (median)=‐44.42; 95% CI=‐50.35,‐38.49; *p* <0.001).

**Conclusion:**

Findings suggest disparities in access to advanced diagnostic investigations and timely initial appointments across rural Australia. These inequalities may preclude access to timely post‐diagnostic services and exacerbate existing barriers to access novel disease modifying therapies which often require advanced diagnostic investigations such as functional neuroimaging.